# Validation of desk-based audits using Google Street View^®^ to monitor the obesogenic potential of neighbourhoods in a pediatric sample: a pilot study in the QUALITY cohort

**DOI:** 10.1186/s12942-022-00301-8

**Published:** 2022-03-26

**Authors:** Jean-Baptiste Roberge, Gisèle Contreras, Lisa Kakinami, Andraea Van Hulst, Mélanie Henderson, Tracie A. Barnett

**Affiliations:** 1 Centre de Recherche du Centre Hospitalier Universitaire Sainte-Justine, 3175 Chemin de la Côte-Sainte-Catherine, Montreal, QC) H3T 1C5 Canada; 2 Faculty of Medicine, Université de Montréal, 2900 Boulevard Édouard-Montpetit, Montreal, QC) H3T 1J4 Canada; 3 Epidemiology and Biostatistics Unit, INRS Institut Armand-Frappier, 531 Boulevard Des Prairies, Laval, QC) H7V 1B7 Canada; 4 Institut de La Statistique du Québec, 1200 Avenue McGill college 5e ÉtageH3B 4J8, Montreal, QC) Canada; 5 Department of Mathematics, Concordia University and PERFORM Centre, 7200 Rue Sherbrooke Ouest, Montreal, QC) H4B 1R6 Canada; 6 Ingram School of Nursing, McGill University, 680 Rue Sherbrooke Ouest #1800, Montreal, QC) H3A 2M7 Canada; 7 Department of Pediatrics, Université de Montréal, 2900 Boulevard Édouard-Montpetit, Montreal, QC) H3T 1J4 Canada; 8 Département de médecine sociale et préventive, École de santé publique de l’Université de Montréal, 5858 Côte-des-Neiges Rd., Montréal, Canada; 9 Department of Family Medicine, McGill University, 5858 Côte-des-Neiges Rd., Montreal, QC) H3S 1Z1 Canada

**Keywords:** Neighbourhood, Pediatric obesity, Built environment, Urban design, Walkability

## Abstract

**Background:**

The suitability of geospatial services for auditing neighbourhood features relevant to pediatric obesity remains largely unexplored. Our objectives were to (i) establish the measurement properties of a desk-based audit instrument that uses Google Street View ^®^ to assess street- and neighbourhood-level features relevant to pediatric obesity (QUALITY-NHOOD tool, the test method) and (ii) comment on its capacity to detect changes in the built environment over an 8-year period. In order to do so, we compared this tool with an on-site auditing instrument (the reference method).

**Methods:**

On-site audits of 55 street- and neighbourhood-level features were completed in 2008 in 512 neighbourhoods from the QUALITY cohort study. In 2015, both repeat on-site and desk-based audits were completed in a random sample of 30 of these neighbourhoods.

**Results:**

Agreement between both methods was excellent for almost all street segment items (range 91.9–99.7%), except for road type (81.0%), ads/commercial billboards (81.7%), road-sidewalk buffer zone (76.1%), and road-bicycle path buffer zone (53.3%). It was fair to poor for perceived quality, safety and aesthetics items (range 59.9–87.6%), as well as for general impression items (range 40.0–86.7%). The desk-based method over-detected commercial billboards and road-sidewalk buffer zone, and generally rated neighbourhoods as less safe, requiring more effort to get around, and having less aesthetic appeal. Change detected over the 8-year period was generally similar for both methods, except that the desk-based method appeared to amplify the increase in the number of segments with signs of social disorder.

**Conclusions:**

The QUALITY-NHOOD tool is deemed adequate for evaluating and monitoring changes in pedestrian- and traffic-related features applicable to pediatric populations. Applications for monitoring the obesogenic nature of neighbourhoods appear warranted.

**Supplementary Information:**

The online version contains supplementary material available at 10.1186/s12942-022-00301-8.

## Background

Close to one third of school-aged children in Canada are overweight or obese and preventative measures are a public health priority [1]. As multiple individual and contextual factors interact to determine obesity in youth [2], evidence supporting the effectiveness of youth obesity prevention programs is needed, notably for home and community-based approaches. [3, 4]

Urban neighbourhood design features are increasingly being considered for their potential to promote healthy lifestyle behaviours in youth, notably those that facilitate active travel to school. Street connectivity and accessibility to local destinations along a safe street network have been found to be positively associated with children's physical activity [5]. For example, walkability [6, 7], street connectivity [6, 8, 9], access to parks, play areas and green spaces [6, 10, 11], and proximity to commercial exercise-related facilities [12] are generally associated with physical activity in children or adolescents. Similarly, results from a recent systematic review concluded that interventions targeting multiple streetscape improvements for walking or cycling, such as crosswalk and sidewalk improvements, improved and covered bike parking, or installation of traffic calming features (textured intersection and zebra crossings for pedestrians), had significant positive impacts on active transportation in children and on physical activity in adults [13].

Many of these neighbourhood-level features are also associated with overweight status in youth [14–18]. However, very few studies in children have assessed area-level *change* in specific features such as park or cycle path access [19, 20]. Documenting the distinct features of neighbourhoods that are most amenable to intervention is an important part of establishing policies designed to address the obesity epidemic. In this pilot study, we systematically scored a comprehensive list of urban design and street-level features, and we repeated this procedure in order to document neighbourhood evolution over an 8-year period. This pilot was conducted in a random subsample of neighbourhoods prior to launching desk-based audits only in the full-scale study.

Sources of information on these features include resident perceptions, topographic maps, and in-person audits using systematic social observation of street segments [21–23]. Combined methods are thought to yield the most comprehensive descriptions of neighbourhoods including both qualitative and fine-grained quantitative aspects [24]; on-site neighbourhood audits, however, can be onerous and broad-scale studies can be time-consuming and costly, while exposing auditors to potential harm or injury [25]. An alternative but equivalent method is warranted, as on-site neighbourhood audits can capture aspects of the built environment that are not available through secondary datasets (notably sidewalks and pavement condition; presence of trash and graffiti; tree canopy characteristics; perceived quality, safety and aesthetics of residences/buildings, yards and decorative features; general impression features of neighbourhoods).

In the last decade, the widespread availability of free internet-based geospatial tools has facilitated novel desk-based or ‘virtual’ methods for assessing urban design and street-level features [25]. One such geospatial tool, *Google Earth*^®^, comprises a software that displays satellite images of the earth's surface, with zoom-in capability at high resolution. *Google Street View*^®^, a feature in *Google Earth*^®^, provides 360° horizontal panoramic views at the street-level, giving the auditor the sensation of ambulating along the street and the capability to inspect the entire street segment with remarkable detail.

In the past five to 10 years, multiple studies have used *Google Street View*^®^, *Google Earth*^®^, or other geospatial tools to characterize environments [25, 26]. Although neighbourhood audits using *Google Street View*^®^ have been reported to be valid and reliable with respect to the assessment of specific neighbourhood features [20, 25–39], little is known about their ability to capture neighbourhood features applicable to pediatric populations [20, 33] (i.e. school corridors, *children at play* signs, etc.) and to our knowledge, none have been used to document changes in neighbourhood features over time. We extend capacity in this area by evaluating a newly developed desk-based audit instrument that exploits *Google Street View*^®^’s data source and its application software. Specifically, we establish its measurement properties, including indices of agreement and performance, by comparing contemporaneous desk-based (test method) and on-site (reference method) assessments. We then describe the occurrence and magnitude of street-level changes and of neighbourhood transformations over an average 8-year period using both our test and reference methods and discuss implications for research.

## Methods

### Study sample

Neighbourhood data were obtained from the QUALITY (QUebec Adipose and Lifestyle InvesTigation in Youth) study, an ongoing longitudinal investigation of the natural history of obesity and related cardiometabolic consequences in youth. A detailed description of the original QUALITY study design and methods is available elsewhere [40]. Briefly, a total of 630 Caucasian families were recruited from 2005 to 2008 via flyers sent to all schools in 3 urban areas of Quebec, Canada; 512 families lived in the greater Montreal area. At least one parent had to be obese at the time of recruitment (based on self-reported height and weight or waist circumference) and the participating child had to be between 8 and 10 years. All family members completed interviewer-administered questionnaires; trained nurses obtained biological, anthropometric, and physiological measurements from children. Follow-ups occurred in 2008–2010 (when children were aged 10–12 years) and again in 2015–2017 (at ages 15 to 17 years). Written informed consent was obtained from both biological parents, and assent was provided by children. The ethics review boards of CHU Sainte-Justine and the Quebec Heart and Lung Institute University approved the study protocol.

### Measures

**Fig. 1:**
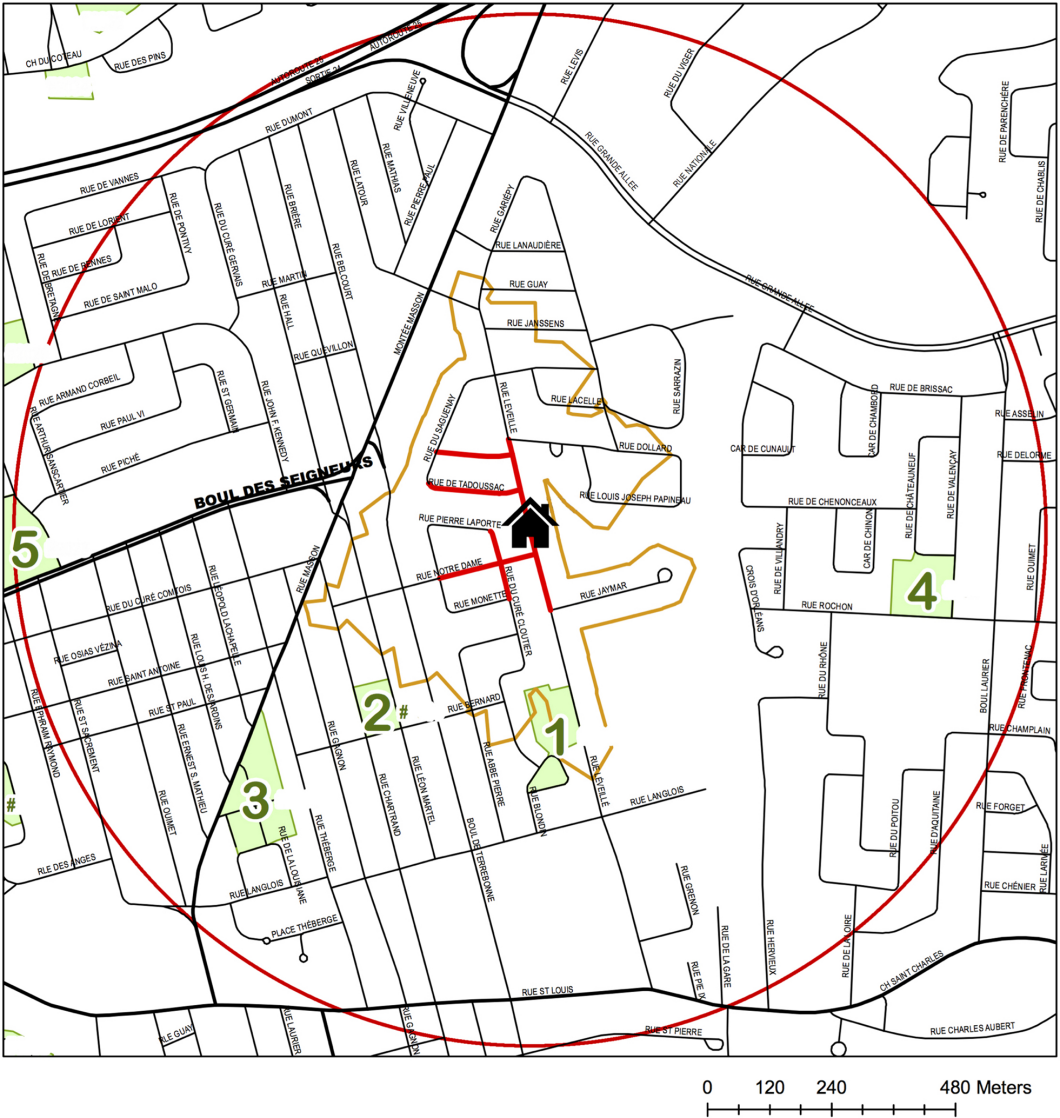
Example of a map used to conduct audits in QUALITY residential neighbourhoods. QUALITY Study, 2008–2015

Systematic social observation (i.e. on-site audits and scoring of street-level elements) was completed at baseline (i.e. 2008) in all 512 Montreal-area neighbourhoods. A geospatial technician produced maps for each address using a spatial database [41], circumscribing the entire 500-m walking network around the family residence (Fig. 1), and highlighting 10 contiguous street segments centered on the residence for detailed evaluation, including all first degree (i.e. connected to the residential) and a random selection of second degree (i.e. connected to a first degree) street segments (Fig. 1). At the 2015 follow-up, 80% of families were still living at the same address and comprised our sampling frame. We conducted a stratified random sample of 30 addresses such that the 30 corresponding neighbourhoods reflected diversity both in area-level socioeconomic status, with median area income ranging from $44,580 to $116,529 CAD per year, and in population density, ranging from less than 2000 to more than 13,000 residents per km^2^.

### Instrument

The original neighbourhood audit tool, called the QUALITY-NHOOD (QUALITY *NeigHbourhood Obesogenic pOtential Diagnosis*), was designed to be used on-site [21]. It was adapted by Dr. Barnett and Dr. Van Hulst in part from existing validated tools [22, 42–45] and underwent extensive pretesting with several teams of trained observers. The final version comprised a total of 55 items, including 46 street-level items and 9 general impression items. Street-level items included land use and design (15 items), street segment installations or signs (13 items), street segment modifications and markings (7 items), and perceived quality, safety and aesthetics (11 items). Items assessing general impressions included neighbourhood safety (3 items), effort required to walk/cycle around the neighbourhood (2 items), presence of nature and green space (1 item), signs of social disorder (1 item), ambiance (1 item), and aesthetics (1 item). To avoid possible confusion between mid-segment zebra crossings, zebra crossing at intersections, textured intersections for pedestrians and pedestrian crossing signs, these were combined and categorized as “pedestrian crossing zone”. Street-level items were scored based on presence or absence, except if otherwise specified. General impression items were scored on a Likert scale, ranging from “not at all” to “a lot”. Documents are available online [46].

The desk-based counterpart to the QUALITY-NHOOD is identical to the original on-site audit tool except for the addition of prompts documenting the date of the *Google Street View*^®^ images for each street segment (see supplementary file). In addition, the *Google Earth*^®^ satellite map provides a useful overhead perspective of the neighbourhood.

In our previous work [47], we characterized neighbourhood types using factor-based scores that captured the presence of any of several specific features on each segment. These included a traffic-calming score (proportion of segments with at least one of the following items: speed bumps, mid-segment stop signs, 30 km/h or lower speed limits, large obstacles, or traffic lights), a pedestrian-facilitating score (proportion of street segments with at least one of the following items: all-ways stop sign, pedestrian crossing zone, and dedicated traffic lights for pedestrians), and a social-disorder score (proportion of street segments with at least one of the following items: any visible trash, graffiti, vacant lots, or abandoned buildings). The three scores were generated using both the desk-based and on-site assessments, and then compared.

### Data collection

Training for the current study involved two observers independently completing both on-site and desk-based audits of 50 street segments in 5 test neighbourhoods with the lead investigator; scoring discrepancies were discussed and resolved until standardization of responses was deemed optimal. For both desk-based and on-site audits, two observers either walked or virtually walked every street segment, using the map to orient themselves; all items were scored after every street segment. Once all items of all 10 segments in a given neighbourhood were scored, the observers walked or virtually walked every street segment in the 500-m walking network around the participant's residence and scored the 10 items pertaining to the general impressions of the neighbourhood. The time needed to complete each on-site and desk-based neighbourhood audit was recorded. Although one observer primarily conducted the desk-based audits, while the other primarily conducted on-site audits, both remained masked with respect to each other’s responses throughout the data collection period.

## Analyses

We computed both exact agreement between corresponding items from the on-site and desk-based audit tools, and Cohen’s kappa, a chance-corrected measure of agreement, as recommended when the goal is to estimate predictive accuracy [48]. A single measure of agreement can be uninformative for variables with substantially skewed distributions, or with considerable variation in bias (i.e. over- or under-detection) [49]. We also computed ICCs for the three factor-based scores. Exact agreement was categorized as excellent (≥ 90%), fair (70 to less than 90%), or poor (< 70%), based on cut-offs used by Aghaabbasi et al. [50] Cohen’s kappa statistic was categorized according to Fleiss as excellent (> 0.75), fair (0.40 to 0.75), or poor (< 0.40) [51]. The desk-based assessments were considered the “test” method, and the on-site assessments were considered the “reference” method. To examine performance of the desk-based method, we tested for asymmetry using the McNemar test for 2 × 2 tables or the Bowker test for 3 × 3 tables. [52] Asymmetry was not computed when there were fewer than 5 discordant observations or when exact agreement was greater than 90%. We also characterized the directionality of discrepancies, i.e. whether there was evidence of potential over- and under-detection when using the desk-based method compared to the on-site method.

To describe street-level change over time, we compared the 2008 and 2015 on-site audits, and documented the frequency of segment-specific street elements and summary scores at both time points. We repeated this approach with the 2015 desk-based method, which we compared with the 2008 on-site method (as no desk-based audit was performed in 2008) and we examined discrepancies. Data were analyzed using STATA software (version 14).

## Results

Out of a possible 300 segments from the sample of 30 neighbourhoods, 295 distinct street segments were retained for analysis. Five street segments were discarded as four were not identified by *Google Street View*^®^ and one was a duplicate segment (i.e., overlapped between two different neighbourhoods). The range of dates on which *Google Street View*^®^ images were captured ranged from June 2009 to October 2015, with 85% captured within the year previous to data collection.

Average time to complete the audit for one neighbourhood (i.e., detailed observations of 10 street segments, scan/brisk walk through the 500 m walking network, and scoring of questionnaire items) was 57 min (range = 31–88 min, SD = 16) for the desk-based audits, and 59 min (range = 45–130 min, SD = 15) for the on-site audits. The median sidewalk width was 148 cm based on the desk-based audit, Vs. 150 cm for the on-site audit.

Estimates of reliability between the scoring of items using the desk-based audit (2015) and those using the on-site audit (2015) appear in Table 1**.** The denominator reflects the total number of street segments audited by both methods (i.e. 295, unless otherwise indicated). Street-level items with a prevalence lower than 5% (for both methods) are not presented in the tables but are listed in the footnote for the sake of transparency.

**Table 1:** Comparison of desk-based and on-site audits of QUALITY residential neighbourhoods in 2015 (n = 295 street segments across 30 neighbourhoods). QUALITY Study, 2008–2015

Characteristic (n = 295 segments)	Exact Agreement with on-site audits (%)^1^	Kappa coefficient or intraclass correlation coefficient^1,3^	Asymmetry^2^ (calculated only when exact agreement < 90%)	
			Significant asymmetry (Yes, No)	Directionality of desk-based reporting
Road and street segment^4^				
*Land use and design*				
Number of street sides available for parking (0, 1, 2)	91.9	0.38		
Number of traffic lanes (1, 2, 3 or more)	93.9	0.70		
Traffic direction (one-way, two-way)	95.3	0.85		
Road type (local street, minor artery, major or industrial artery)	81.0	0.71	Yes (p < 0.001)	Scores roads as busier
Number of street sides with a sidewalk (0, 1, 2)	94.2	0.93		
Public transportation available (present/absent)	97.3	0.90		
Predominantly residential (yes/no)	95.9	0.70		
Back alleys (index street segment only) (present/absent)	93.3	0.76		
Exterior playgrounds or fields (present/absent)	96.3	0.72		
Any restaurant (present/absent)^5^	97.9	0.59		
Convenience/corner store (present/absent)	94.2	0.67		
Ads/commercial billboards (present/absent)	81.7	0.17	Yes (p < 0.0001)	Reports more
*Street segment installations or signs (present/absent)*				
Traffic lights for pedestrians	97.6	0.78		
Traffic lights for cars	98.0	0.92		
All-ways stop sign	94.2	0.88		
Pedestrian crossing zone^6^	93.6	0.71		
School corridor	93.2	0.76		
30 km/h speed limit	96.6	0.84		
“Watch out for children”/“Children playing”/Neighbourhood watch signs	91.9	0.62		
*Street segment modifications and markings (present/absent)*				
Intersection choker^7^	98.6	0.88		
Speed bump	99.7	0.95		
Road-sidewalk buffer zone (of segments with sidewalks)^8^	76.1	0.30	Yes (p < 0.0001)	Reports more
Bicycle path	97.3	0.70		
Road-bicycle path buffer zone^8^ (of segments with bicycle paths)	53.3	–	No (p = 0.450)	N/A
*Perceived quality, safety and aesthetics*				
Deteriorated sidewalks (Yes/No)	69.4	0.27	Yes (p = 0.0002)	Reports less
Deteriorated pavement (Yes/No)	74.7	0.51	Yes (p < 0.0001)	Reports less
Trash (present/absent)	75.6	0.03	Yes (p < 0.0001)	Reports more
Graffiti (present/absent)	85.1	0.55	Yes (p = 0.0015)	Reports less
Tree canopy^9^	59.9	0.34	Yes (p < 0.0001)	Reports fewer trees
Well-maintained residences/buildings (all or almost all/about ¾/about half or less)	87.6	0.47	Yes (p < 0.0001)	Reports lower proportion
Well-maintained front yards (all or almost all / about ¾/about half or less)	68.6	0.29	Yes (p = 0.0006)	Reports higher proportion
Buildings with decorative features^10^ (all or almost all / about ¾/about half or less)	61.9	0.40	Yes (p < 0.0001)	Reports lower proportion
Summary variables^11^				
> 1 traffic calming measure	93.9	0.93		
> 1 measure to facilitate pedestrians	93.6	0.95		
> 1 signs of social disorder	78.9	0.62	Yes (p < 0.0001)	Reports more signs
General impression (n = 30 neighbourhoods)				
*Safety from vehicular traffic for pedestrians* (safe/a little, quite, very unsafe)	50.0	0.06	Yes (p = 0.0352)	Reports less safe
*Safety from vehicular traffic for cyclists* (safe/a little, quite, very unsafe)	70.0	0.33	No (p = 0.7839)	
*Effort required to get around on foot* (none/any)^12^	40.0	0.06	Yes (p < 0.0001)	Reports more effort
*Effort required to get around by bicycle* (none/any)^12^	46.7	0.05	Yes (p < 0.0001)	Reports more effort
*Overall neighbourhood safety* (very/mostly, somewhat, not at all)	86.7	0.69	No (p = 0.1250)	
*Natural spaces* (few/many)	63.3	0.28	Yes (p = 0.0009)	Reports fewer natural spaces
*Signs of social disorder* (none/any)	76.7	0.44	No (p = 0.2568)	
*General ambiance* (very, quite pleasurable/more or less, not at all pleasurable)^13^	86.7	0.37		
*General aesthetics* (very, quite appealing/more or less, not at all appealing)	80.0	0.39	Yes (p = 0.0143)	Reports more appeal

^1^The denominator is the total number of street segments audited by the on-site method (n = 295 for most items). Due to missing data, the denominator is 290 for buildings with decorative features, 293 for presence of bike lanes, and 294 for both condition of pavement and well-maintained front yards. Values in red indicate poor agreement; values in green indicate fair agreement

^2^McNemar test of asymmetry for 2 × 2 tables, Bowker test for 3 × 3 tables; it was not computed when there were fewer than 5 discordant items (i.e. *b* + *c* less than 5) or agreement was greater than 90%

^3^For 2 × 2 tables, simple Kappa was reported. For ordinal variables with more than 2 categories, weighted Kappa was reported. Kappa could not be calculated when there were too many missing data. Intraclass correlation coefficients were reported instead of Kappa coefficients for the three summary variables

^4^The following items were not included in the table due to low frequency (below 5% for both methods) for the following: mid-segment stop sign, bicycle-sharing station, median or island (note: 12 scored with desk-based, 3 with on-site), large obstacle, signs of vandalism, condemned building, sports complex, and adequate street lights. Sidewalk width is also not included in this table

^5^This items includes: regular restaurants, fast food restaurants, and coffee shops

^6^This item includes: midsegment zebra crossing, zebra crossing at the intersection, textured intersection for pedestrians, and pedestrian crosswalk sign

^7^Build-outs added to a road at or near the intersection to narrow it

^8^Categories are as follow: none / buffer zone without visual obstruction, buffer zone with visual obstruction, obstruction only

^9^Density of trees on the street segment and extent of the shade they create (not at all or a few / a few but isolated or only on one street side or not creating much shade / many)

^10^Decorative features refer to items that are meant to embellish the outdoor spaces. Examples include, but are not limited to plants, flowers, well-kept bushes and decorative objects

^11^The traffic calming measures include speed bumps, mid-segment stop signs, 30 km/h or lower speed limits, large obstacles, or traffic lights. The pedestrian-facilitating measures include all-ways stop sign, pedestrian crossing zone [4], and dedicated traffic lights for pedestrians. Signs of social disorder include any visible trash, graffiti, vacant lots, or abandoned buildings. Intraclass correlation coefficients are reported

^12^For effort to get around, any effort includes: a little effort / much, and a great deal of effort

^13^Asymmetry was not computed here as *b* + *c* was less than 5

Exact agreement was excellent (≥ 90%) for almost all *street segment land use and design items*, *street segment installations or signs*, *street segment modifications and markings*. Exact agreement was fair (70 to less than 90%) for *road type, ads/commercial billboards,* and *road-sidewalk buffer zone*, and poor (< 70%) for the *road-bicycle path buffer zone* (computed for the 10 segments with bicycle paths). Exact agreement for *perceived quality*, *safety and aesthetics* items was poor to fair, ranging from 59.9% (*extent of tree canopy*) to 87.6% (*proportion of well-maintained residences/buildings: all or almost all/about ¾/about half or less*). Exact agreement was excellent for the summary variables related to traffic calming and pedestrian facilitators, and fair for social disorder. Exact agreement for general impression items was also poor to fair, ranging from 40.0% (*effort required to get around on foot: none/any*) to 86.7% (*overall neighbourhood safety: very/mostly, somewhat, not at all;* and *general ambiance: very, quite pleasurable/more or less, not at all pleasurable*). Agreement based on Cohen’s kappa was generally consistent with those based on exact agreement, except for variables with substantially skewed distributions; in these circumstances, kappa is not a recommended measure of reliability [49]. We only audited back alleys for the 4 residential segments that had one (out of a possible 30 index street segments). Due to this low frequency, no meaningful performance measures could be estimated and quantitative analyses for back alleys are not reported.

Based on the tests of asymmetry (i.e. the McNemar or Bowker tests), reporting discrepancies between the desk-based and on-site methods were observed for *road type, ads/commercial billboards* and *road-sidewalk buffer zone*. The desk-based method over-detected presence or frequency in comparison to the on-site method in these three cases. All items pertaining to perceived quality, safety and aesthetics were asymmetric; although no systematic over or under detection was evident, items that were under-reported by the desk-based method included *deterioration of sidewalks and pavement, presence of graffiti, extent of tree canopy, well-maintained residences/buildings,* and *presence of decorative features*. Of the three neighbourhood-level factor-based scores (traffic-calming, pedestrian-facilitation, social-disorder), only the social disorder score was asymmetric, with more signs of social disorder detected with the desk-based method. Finally, with respect to general impression items, desk-based audits rated neighbourhoods as less safe for pedestrians, requiring more effort to get around on foot or by bicycle, having fewer natural spaces, and having less aesthetic appeal, in comparison to on-site audits; no asymmetry was observed for the other general impression items.

Table 2 describes changes detected over time, between 2008 and 2015, as observed using the 2015 on-site and desk-based audits. In all cases, the denominator is 287, corresponding to the number of segments that could be definitively matched from 2008 to 2015 and that were audited using both methods. Regardless of the method used, substantial increases (≥ 40% difference) between 2008 and 2015 were detected in school corridors and in intersection chokers, and substantial declines in 30 km/h speed limit signs and *“Watch out for children”/ “Children playing”/Neighbourhood Watch* signs. A modest decrease (15 to less than 40% difference) was detected in traffic lights for pedestrians. Other changes over time for specific features were small in magnitude (5 to less than 15% difference), with only slight discrepancies between methods. The only exception was for pedestrian crossing zone, for which a small decrease was reported using the on-site method and a small increase was reported using the desk-based method. Change observed using the summary scores pointed to a modest decrease in segments with any traffic-calming measures and a slight increase in segments with any pedestrian-facilitating measures, with both methods performing almost identically. On the other hand, change was discordant with respect to segments with any signs of social disorder, with a substantial increase in the number signs of social disorder detected using the desk-based method, and only a slight increase using the on-site method.

**Table 2:** Street-level change in QUALITY residential neigbourhoods between 2008–2015 using both desk-based and on-site audits (n = 287 Montreal street segments). QUALITY Study, 2008–2015

	Present in 2008 (on-site audits) n	Present in 2015 (on-site audits) n	Absolute Difference (% difference) based on 2008 and 2015 on-site audits	Present in 2015 (desk-based audits) n	Absolute Difference (% difference) based on 2008 on-site and 2015 desk-based audits
Traffic lights for pedestrians	21	14	− 7 (− 33.3)	17	− 4 (− 19.0)
Traffic lights for cars	38	39	+ 1 (+ 2.6)	41	+ 3 (+ 7.9)
All-ways stop signs	112	122	+ 10 (+ 8.9)	121	+ 9 (+ 8.0)
School corridor	8	46	+ 38 (+ 475.0)	56	+ 48 (+ 600.0)
Intersection choker^1^	11	18	+ 7 (+ 63.6)	18	+ 7 (+ 63.6)
Pedestrian crossing zone^2^	39	35	− 4 (− 10.3)	42	+ 3 (+ 7.7)
30 km/h speed signs	58	30	− 28 (− 48.3)	34	− 24 (− 41.4)
“Watch out for children”/“Children playing”/neighbourhood watch signs	68	31	− 37 (− 54.4)	27	− 41 (− 60.3)
> 1 traffic calming measure^3^	99	63	− 36 (− 36.4)	68	− 31 (− 31.3)
> 1 measure to facilitate pedestrians^4^	142	152	+ 10 (+ 7.0)	157	+ 15 (+ 10.6)
> 1 sign of social disorder^5^	61	65	+ 4 (+ 6.6)	90	+ 29 (+ 47.5)

^1^Build-outs added to a road at or near the intersection to narrow it

^2^This item includes: mid-segment zebra crossing, zebra crossing at the intersection, textured intersection for pedestrians, and pedestrian crosswalk sign

^3^The traffic calming measures include speed bumps, mid-segment stop signs, 30 km/h or lower speed limits, large obstacles, or traffic lights

^4^The pedestrian-facilitating measures include all-ways stop sign, pedestrian crossing zone, and dedicated traffic lights for pedestrians

^5^Signs of social disorder include any visible trash, graffiti, vacant lots, or abandoned buildings

## Discussion

Our aim was to estimate the measurement properties of a desk-based instrument designed to assess the obesogenic potential of neighbourhoods remotely, and to explore its capacity to monitor neighbourhood change over time. To our knowledge, the QUALITY-NHOOD tool is the first such instrument specifically designed to assess a wide spectrum of youth-relevant street-level features, notably those that facilitate safe, active travel to school. We found that the desk-based instrument performed as well as on-site audits in most situations.

Agreement was generally excellent, especially for relatively permanent items that are less seasonally variable. Exceptions included items that were of relatively low prevalence. Agreement was however poor to fair for items that were more qualitative in nature, variable or dynamic such as the presence of trash, the presence of decorative features and safety from traffic; this is consistent with other studies that reported lower levels of agreement for items requiring subjective appraisal [25, 27, 36]. Clearly, the presence of trash will vary depending on collection days and on whether debris was left behind; on-site audits have the advantage of partially taking into account arbitrary and seasonal variation through repeated measures, for example by repeating audits three times over nine months, an option not available using the desk-based method. Although items that are seasonally variable or highly subjective in nature may not be appropriate for desk-based assessment, *Google Street View*^®^ appears to be an efficient tool for collecting data on street design- and road network features.

Our findings support the use of desk-based audits to monitor neighbourhood features with respect to their obesogenic potential over time. We detected a modest decline in traffic-calming measures, largely due to the removal of 30 km/h speed limit signs. Although there was a modest overall increase in pedestrian- and school-travel-friendly features, notably due to the increase in school corridors and in intersection chokers, we also noted a decrease in dedicated traffic lights for pedestrians. Due to the possible confusion in certain types of signs (i.e. between “Watch out for children”, “Children playing” and neighbourhood watch signs), changes may be artifactual and no firm conclusions can be made with respect to signs. Desk-based audits were reliable for assessing the food environment, but there were too few changes over time to comment on its suitability for monitoring changes in the food environment over time in our sample; replication in larger samples is warranted. Our study also suggests that in an urban setting like Montreal, changes occurring within 8 years and possibly over an even shorter time span can easily be detected using *Google Street View*^®^. Moreover, specific key items, such as speed limit signs and marking, could reasonably be monitored on a yearly basis, or as images are updated.

Thus, while change detected over time was generally similar for both methods (i.e. 2008 on-site and 2015 on-site Vs. 2008 on-site and 2015 desk-based), it was notably discrepant for pedestrian crossing zones. If the on-site method is considered to be more valid, then our findings suggest that desk-based monitoring of street signs tends to perform poorly. Performance of the desk-based method may have been compromised if portions of images were obstructed by foliage or vehicles, thereby limiting visual interpretation, or if signs were offset from sidewalks. It is also possible that *Google Street View*^®^’s resolution was inadequate to discern smaller street signs, yielding misclassification for sign-type. In addition, while 85% of *Google Street View*^®^ were captured in the past year (2014–2015), 15% were dated prior to 2014, and in rare instances dated back to 2009, potentially pre-dating actual changes documented on-site. Nevertheless, the two auditing methods agreed with respect to the directionality of change in almost all instances; in the case of the discordant item (pedestrian crossing zone), the magnitude of the discrepancy was small.

It should be considered that in some instances, the desk-based audit may have performed better than the on-site audit, for example by reducing distractions, allowing for a more systematic audit, facility to double-check, and a unique overhead perspective. Moreover, although time to complete audits was similar, using desk-based audits reduces travel time and expense, as well as potential for harm and injury. Although we selected the on-site audit as the reference method and concluded that it performed better for assessing and monitoring signs of social disorder, apparent over-reporting when using the desk-based may suggest it to be a more sensitive method. Expanded audits using different designs may elucidate this.

Strengths of our study include the novelty, the large number of items measuring a variety of neighbourhood characteristics of relevance to children, as well as an extensive and rigorous training period for auditors. Moreover, the inter- and intra-reliability of the on-site audits had been previously established [21]. Some limitations, however, should be noted. First, this study was conducted in a single metropolitan area; generalizability to different urban centers is unknown. However, neighbourhoods were diverse with respect to area-level socioeconomic status and population density, and some segment-specific items are likely to be universal. Second, while geospatial coverage of *Google Street View*^®^ is broad and considerable, it is not geographically complete: indeed, images were only available in areas accessible to cars. The on-site audits had included park assessments [53], a component not yet possible with the desk-based method. Furthermore, while we used the most recent *Google Street View*^®^ images available at the time of data collection, the range of dates on which the images were captured (2009 to 2015) might have introduced a selection bias. As 85% of the images were captured within the year previous to data collection, we consider this possible bias as minimal.

## Conclusion

We conclude overall that the QUALITY-NHOOD desk-based audit tool is adequate for evaluating and monitoring changes in pedestrian- and traffic-related features applicable to pediatric populations. Neighbourhood-level variation in obesity has been well established, but specific targets for intervention are less clear. Designing safe and connected walking and cycling infrastructure is a key component of neighbourhoods prioritizing the promotion of more physically active lifestyles. Identification of additional salient obesogenic features remain a research priority, but evaluating the impact of simple modifications in urban design is warranted, as is determining if the addition of some features can compensate for the loss of others. Accurate audits of neighbourhoods would allow us to monitor the magnitude of changes and the effectiveness of interventions, both of which are needed to inform policy. The QUALITY-NHOOD tool is a valid and feasible method for assessing street-level features of the built environment that may influence obesity by promoting or hindering active lifestyles; it may also be considered to monitor the food environment. Future analyses will incorporate changes in associated lifestyle behaviours, providing a strong inferential basis with which to identify targets that are not only amenable to change through policy or other measures, but that are also the most salient for health.

## Supplementary Information

**Additional file 1.** Street segment form (46 items).

## Data Availability

The datasets used and/or analysed during the current study are available from the corresponding author upon reasonable request.

## Abbreviations

QUALITY: QUebec Adipose and Lifestyle InvesTigation in Youth
NHOOD: NeigHbourhood Obesogenic pOtential Diagnosis
SD: Standard deviation
