# Correction: Validation of desk‑based audits using Google Street View^®^ to monitor the obesogenic potential of neighbourhoods in a pediatric sample: a pilot study in the QUALITY cohort

**DOI:** 10.1186/s12942-022-00303-6

**Published:** 2022-06-02

**Authors:** Jean‑Baptiste Roberge, Gisèle Contreras, Lisa Kakinami, Andraea Van Hulst, Mélanie Henderson, Tracie A. Barnett

**Affiliations:** 1 Centre de Recherche du Centre Hospitalier, Universitaire Sainte-Justine, 3175 Chemin de la Côte-Sainte-Catherine, Montreal, QC H3T 1C5 Canada; 2 Faculty of Medicine, Université de Montréal, 2900 Boulevard Édouard-Montpetit, Montreal, QC H3T 1J4 Canada; 3 Epidemiology and Biostatistics Unit, INRS Institut Armand-Frappier, 531 Boulevard Des Prairies, Laval, QC H7V 1B7 Canada; 4 Institut de La Statistique du Québec, 1200 Avenue McGill College 5e Étage, Montreal, QC H3B 4J8 Canada; 5 Department of Mathematics, Concordia University and PERFORM Centre, 7200 Rue Sherbrooke Ouest, Montreal, QC H4B 1R6 Canada; 6 Ingram School of Nursing, McGill University, 680 Rue Sherbrooke Ouest #1800, Montreal, QC H3A 2M7 Canada; 7 Department of Pediatrics, Université de Montréal, 2900 Boulevard Édouard-Montpetit, Montreal, QC H3T 1J4 Canada; 8 Département de médecine Sociale et Préventive, École de Santé Publique de l’Université de Montréal, 5858 Côte-des-Neiges Rd., Montreal, Canada; 9 Department of Family Medicine, McGill University, 5858 Côte-des-Neiges Rd., Montreal, QC H3S 1Z1 Canada

## Correction to: International Journal of Health Geographics (2022) 21:2 10.1186/s12942-022-00301-8

In this article, revised Fig. 1 has been replaced with a map with a more appropriate resolution. The original article [1] has been corrected.

**Fig. 1:**
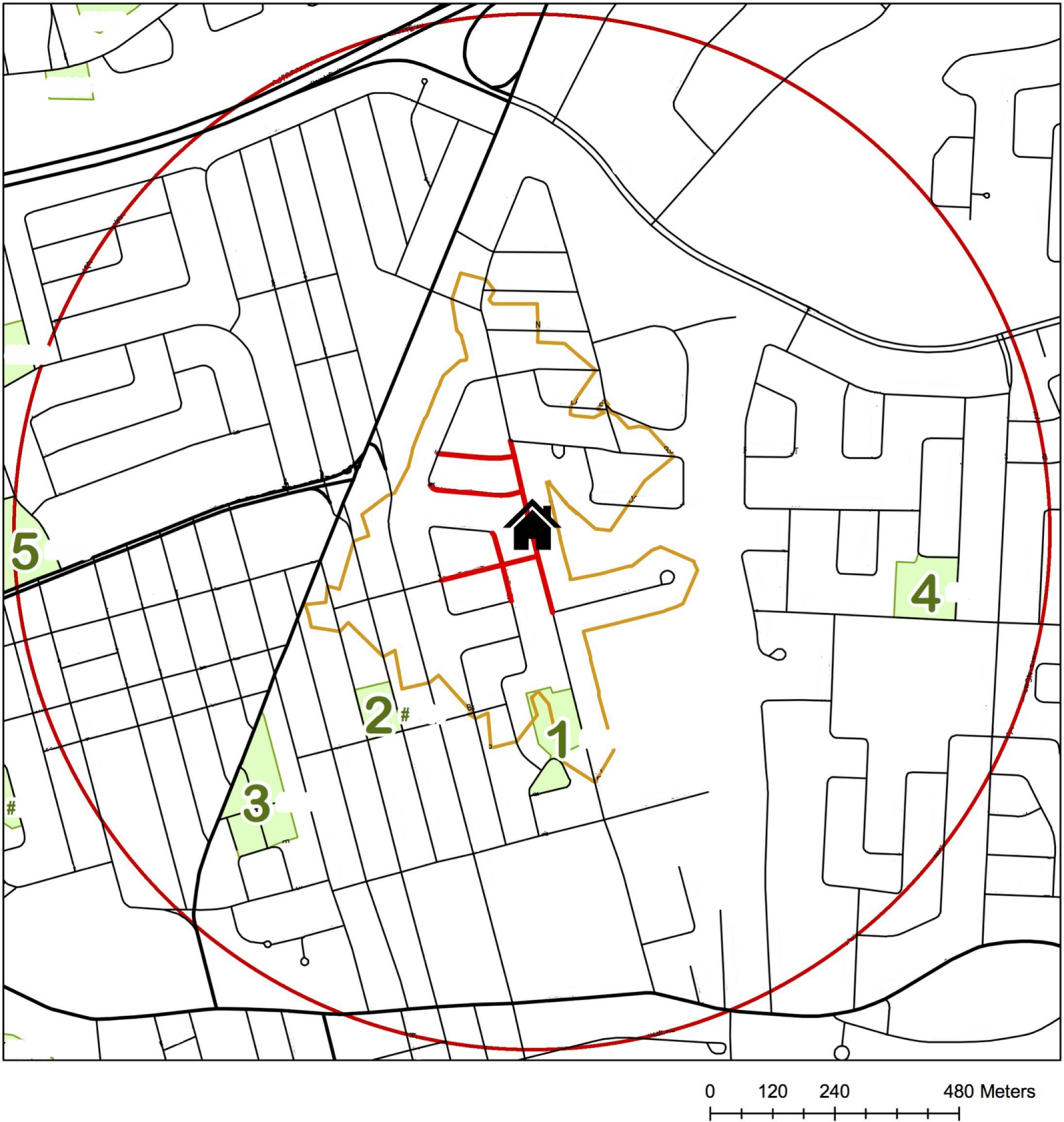
Example of a map used to conduct audits in QUALITY residential neighbourhoods. QUALITY Study, 2008–2015
