# Supplementary material for: Validation of desk-based audits using Google Street View® to monitor the obesogenic potential of neighbourhoods in a pediatric sample: a pilot study in the QUALITY cohort
Source: Int J Health Geogr. 2022 Mar 26;21:2. doi: 10.1186/s12942-022-00301-8 (PMC8961916; doi:10.1186/s12942-022-00301-8)
Supplement: Supplementary file 1 — Additional file 1. Street segment form (46 items). [file 12942_2022_301_MOESM1_ESM.docx]

**QUALITY-NHOOD tool**

A desk-based audit instrument that uses Google Street View ®

to assess street- and neighbourhood-level features relevant to pediatric obesity

**STREET SEGMENT FORM (46 ITEMS)**

10 forms/neighbourhood (including index street segment)

| *Identification* **(PLEASE COMPLETE)** |
| --- |
| Observer ID: __________  Neighbourhood ID: __________  Street Segment ID: __________ Index segment **(PLEASE CIRCLE)**: yes, no  Day of observation: __________  Date of Google Street View ® images: __________  Observation Starting Time (00:00): __________  Observation Ending Time (00:00): __________ |

| *Sidewalk width* **(PLEASE COMPLETE)** |
| --- |
| __________ cm |

| *Land use and design* **(PLEASE CIRCLE)** | | | | | | | | | |
| --- | --- | --- | --- | --- | --- | --- | --- | --- | --- |
| Number of street sides available for parking | | 0 | | 1 | | | | 2 | |
| Number of traffic lanes | | 1 | 2 | | | | 3 | | More |
| Traffic direction | | One-way | | | | | Two-way | | |
| Road type | | Local street | Minor artery | | | | Major artery | | Industrial artery |
| Number of street sides with a sidewalk | | 0 | | 1 | | | | 2 | |
| Public transportation available | | Present | | | | | Absent | | |
| Predominantly residential | | Yes | | | | | No | | |
| Back alleys (index street segment only) | | Present | | | | | Absent | | |
| Exterior playgrounds or fields | | Present | | | | | Absent | | |
| Regular restaurants | | Present | | | | | Absent | | |
| Fast food restaurants | | Present | | | | | Absent | | |
| Coffee shops | | Present | | | | | Absent | | |
| Convenience/corner store | | Present | | | | | Absent | | |
| Sports complex | | Present | | | | | Absent | | |
| Ads/commercial billboards | | Present | | | | | Absent | | |
| *Street segment installations or signs* **(PLEASE CIRCLE)** | | | | | | | | | |
| Traffic lights for pedestrians | | Present | | | Absent | | | | |
| Traffic lights for cars | | Present | | | Absent | | | | |
| All-ways stop sign | | Present | | | Absent | | | | |
| Mid-segment stop sign | | Present | | | Absent | | | | |
| Midsegment zebra crossing | | Present | | | Absent | | | | |
| Zebra crossing at the intersection | | Present | | | Absent | | | | |
| Textured intersection for pedestrians | | Present | | | Absent | | | | |
| Pedestrian crosswalk sign | | Present | | | Absent | | | | |
| Median or island | | Present | | | Absent | | | | |
| Large obstacle | | Present | | | Absent | | | | |
| School corridor | | Present | | | Absent | | | | |
| 30 km/h speed limit | | Present | | | Absent | | | | |
| “Watch out for children” / “Children playing” / Neighbourhood watch signs | | Present | | | Absent | | | | |
| Bicycle sharing station | | Present | | | Absent | | | | |
| *Street segment modifications and markings* **(PLEASE CIRCLE)** | | | | | | | | | |
| Intersection choker^1^ | | Present | | | | Absent | | | |
| Speed bump | | Present | | | | Absent | | | |
| Road-sidewalk buffer zone (of segments with sidewalks) | | Present^2^ | | Absent | | | | N/A^3^ | |
| Bicycle path | | Present | | | | Absent | | | |
| Road-bicycle path buffer zone (of segments with bicycle paths) | | Present^2^ | | Absent | | | | N/A^3^ | |
| *Perceived quality, safety and aesthetics* **(PLEASE CIRCLE)** | | | | | | | | | |
| Deteriorated sidewalks | Yes | | | | | | No | | |
| Deteriorated pavement | Yes | | | | | | No | | |
| Trash | Present | | | | | | Absent | | |
| Graffiti | Present | | | | | | Absent | | |
| Signs of vandalism | Present | | | | | | Absent | | |
| Condemned building | Present | | | | | | Absent | | |
| Adequate street lights | Present | | | | | | Absent | | |
| Tree canopy | Not at all or a few | | | A few but isolated or only on one street side or not creating much shade | | | | Many | |
| Well-maintained residences/buildings | All or almost all | | | About ¾ | | | | About 1/2 or less | |
| Well-maintained front yards | All or almost all | | | About ¾ | | | | About 1/2 or less | |
| Buildings with decorative features^4^ | All or almost all | | | About ¾ | | | | About 1/2 or less | |

^1^ Build-outs added to a road at or near the intersection to narrow it.

^2^ Buffer zones include: buffer zone without visual obstruction, buffer zone with visual obstruction, obstruction only.

^3^ N/A means “not applicable”.

^4^ Decorative features refer to items that are meant to embellish the outdoor spaces. Examples include, but are not limited to plants, flowers, well-kept bushes and decorative objects.

*Tool developed by: Jean-Baptiste Roberge, Gisèle Contreras, Lisa Kakinami, Andraea Van Hulst, Mélanie Henderson, and Tracie A. Barnett. The original neighbourhood audit tool was designed to be used on-site. It was adapted by Andraea Van Huls and Tracie Barnett in part from existing validated tools.*

**QUALITY-NHOOD tool**

A desk-based audit instrument that uses Google Street View ®

to assess street- and neighbourhood-level features relevant to pediatric obesity

**NEIGHBOURHOOD FORM (9 ITEMS)**

1 form/neighbourhood

| *Identification* **(PLEASE COMPLETE)** |
| --- |
| Observer ID: __________  Neighbourhood ID: __________  Street Segment ID: __________  Day of observation: __________  Date of Google Street View ® images: __________  Observation Starting Time (00:00): __________  Observation Ending Time (00:00): __________ |

| *General impression* (**PLEASE CIRCLE)** | | |
| --- | --- | --- |
| Safety from vehicular traffic for pedestrians | Safe | A little, quite, very unsafe |
| Safety from vehicular traffic for cyclists | Safe | A little, quite, very unsafe |
| Effort required to get around on foot^1^ | None | Any |
| Effort required to get around by bicycle^1^ | None | Any |
| Overall neighbourhood safety | Very safe | Mostly, somewhat, not at all safe |
| Natural spaces | Many | Few |
| Signs of social disorder | None | Ant |
| General ambiance | Very, quite pleasurable | More or less, not at all pleasurable |
| General aesthetics | Very, quite pleasurable | More or less, not at all pleasurable |

^1^ For effort to get around, any effort includes: a little effort / much, and a great deal of effort.

*Tool developed by: Jean-Baptiste Roberge, Gisèle Contreras, Lisa Kakinami, Andraea Van Hulst, Mélanie Henderson, and Tracie A. Barnett. The original neighbourhood audit tool was designed to be used on-site. It was adapted by Andraea Van Huls and Tracie Barnett in part from existing validated tools.*
